# Prediction of smartphone overdependence and analysis of its influencing factors among older adults based on machine learning

**DOI:** 10.3389/fpsyg.2026.1704618

**Published:** 2026-01-27

**Authors:** Haiyan Kong, Xinyu Wang, Hualong Fang, Xuming Shangguan

**Affiliations:** 1 School of Business, Xinyang Normal University, Xinyang, China; 2 Dabie Mountain Economic and Social Development Research Center, Xinyang, China; 3 Department of Management Information Systems, Chungbuk National University, Cheongju, Republic of Korea; 4 Department of Global Business, Chungbuk National University, Cheongju, Republic of Korea

**Keywords:** digital behavior, machine learning, older adults, risk factors, smartphone overdependence

## Abstract

**Background:**

With the widespread use of smartphones among middle-aged and older adults, the risks associated with excessive use and dependence on smartphones have become increasingly apparent. This study aims to identify and predict the risk factors for smartphone overdependence among older adults in South Korea, utilizing machine learning methods to construct predictive models.

**Methods:**

We utilized panel data from the “2023 Smartphone Overdependence Survey” provided by the National Information Society Agency (NIA) of South Korea. This study specifically focuses on the older adult population aged 60 and above, identifying key factors influencing their smartphone overdependence. A variety of machine learning-based binary classifiers were evaluated, including XGBoost, SVM, LR, KNN, DT, and NB. Their predictive accuracy and performance were compared comprehensively. Model performance was assessed using multiple metrics, including confusion matrix, accuracy, precision, recall, F1 score, and AUC.

**Results:**

The XGBoost classifier performed the best in predicting smartphone overdependence among older adults, with an accuracy of 0.925. Through feature importance analysis, we found that demographic characteristics, time composition of smartphone use, awareness of smartphone overdependence problem, and content of smartphone use were the main influencing factors in predicting smartphone overdependence among older adults.

**Conclusion:**

Artificial intelligence algorithms have the potential for predictive and explanatory capabilities, identifying the risk of smartphone overdependence among older adults and the associated risk factors. This has significant theoretical and practical implications for understanding and addressing this issue.

## 1 Introduction

With the accelerating development of the information society, the dual processes of digitalization and population aging are becoming increasingly intertwined, fostering the integration of older adults into a digital media environment centered around smart devices. In recent years, both the frequency and intensity of smartphone use among older adults have increased significantly, a trend that has become particularly prominent on a global scale. A growing body of research has demonstrated that smartphone use can, to a certain extent, alleviate loneliness, enhance subjective well-being, improve social connectedness, and promote overall quality of life among older adults (Szabo et al., 2019; Liu et al., 2022; Soundararajan et al., 2023; Toh and Khoo, 2025). As such, smart devices are increasingly regarded as critical tools in advancing practices of active aging and digital empowerment, offering older individuals greater access to information and opportunities for social interaction.

However, the deepening digital engagement has also introduced new risks and vulnerabilities. As the duration and frequency of smartphone use increase, the associated negative consequences have become more pronounced, particularly in relation to physical and cognitive domains such as vision, sleep quality, and attention span. These issues have gradually extended beyond the technological dimension and manifested as psychological and behavioral forms of dependency (Long et al., 2017; Alhassan et al., 2018; Gezgin, 2018; Jeong and Oh, 2020). Smartphone overdependence refers to a pattern of persistent, repetitive, and prioritized smartphone use despite the presence of adverse consequences for health, social relationships, or functional well-being, accompanied by a high level of cognitive and behavioral reliance on the device (Kim S. H. et al., 2024). According to annual monitoring data from the National Information Society Agency (NIA) of South Korea, as of 2022, approximately 17.5% of individuals aged 60 and above were classified as being at either “high risk” or “potential risk” of smartphone overdependence—an increase from 16.8% in 2020—underscoring the growing digital vulnerability among the older population (National Information Society Agency, 2023).

The integration of digitalization and aging has become a significant trend in contemporary international society. In recent years, there has been a sharp increase in smartphone usage among older adults. However, with the overuse of smartphones, negative impacts such as physical and psychological issues have gradually emerged, making smartphone overdependence a prominent social problem (Alhassan et al., 2018; Gezgin, 2018; Jeong and Oh, 2020). Smartphone overdependence refers to the excessive use of smartphones despite their negative impacts, making them more important than other activities in daily life (Kim S. H. et al., 2024). According to a survey by the National Information Society Agency (National Information Society Agency, 2023), the proportion of people at risk of smartphone overdependence among those aged 60 and above was 17.5% as of 2022, a significant increase from 16.8% in 2020. Considering that the total number of people at risk of smartphone overdependence was 24.2% in 2021, this proportion is particularly notable. Similarly, older adults also find it difficult to escape smartphone overdependence, especially after the age of 60, due to the reduction of social roles and weakening of offline relationships, which may increase smartphone usage and thus elevate the risk of smartphone overdependence.

Although the prevalence of smartphone overdependence among older adults is lower than that of high-frequency user groups such as adolescents, its consequences tend to be more severe and potentially irreversible. On one hand, the functional substitution effect of smartphone use may further diminish older adults’ motivation for offline social engagement and participation in daily life. On the other hand, excessive dependence may exacerbate their structural vulnerabilities in areas such as cognitive decline, physical health deterioration, misinformation susceptibility, and exposure to digital fraud (Jeong et al., 2020; Meshi et al., 2020). Within the coexisting context of “technological late adoption” and “emotionally compensatory use,” older adults are more likely to rely on smartphones as key tools for emotional regulation and maintaining social connections. This results in a psychologically driven and compensatory attachment to digital media. Consequently, while the digital devices have brought unprecedented convenience to daily life, it has also revealed emerging dependence risks closely linked to “digital vulnerability.” These findings underscore the urgent need to develop risk identification frameworks and intervention strategies tailored to the characteristics of older users, in order to achieve a truly inclusive and healthy digital aging transition.

In recent years, there has been a growing body of research examining smartphone overuse and its psychosocial consequences. Systematic reviews and meta-analyses have indicated that the overall prevalence of smartphone overdependence in the general population—including adolescents, university students, and younger to middle-aged adults—is approximately 14% (Meng et al., 2022). Empirical studies have consistently shown that excessive smartphone use is associated with a range of adverse health outcomes, including depression, loneliness, sleep disturbances, and reduced subjective well-being (Özbek and Karaş, 2022; Wang et al., 2024). In parallel, scholars have developed explanatory models from the perspectives of individual psychological traits—such as anxiety and self-control—as well as the structure of social support systems (Bertocchi et al., 2022). This growing research trend highlights smartphone overdependence as an emerging risk behavior in digital society, exerting profound impacts on individuals’ mental health and social functioning.

However, it is noteworthy that existing studies have predominantly focused on adolescents and younger to middle-aged adults, particularly samples under high academic pressure or in digitally saturated environments, such as university students (Meshi et al., 2020; Rochat et al., 2021; Zhang et al., 2021). This population bias not only limits our understanding of smartphone dependence across different stages of the life course, but also overlooks the unique vulnerabilities and behavioral heterogeneity exhibited by older adults during the process of digital inclusion.

Unlike younger generations who have grown up in digitally native environments, older adults are typically introduced to digital technologies during midlife or after retirement, often lacking a systematic path of technology adoption. Their usage motivations tend to center around emotional connection, safety assurance, and informational compensation (Kebede et al., 2022; Schroeder et al., 2023). At the same time, factors such as weakened social roles, reduced offline social engagement, and declining cognitive regulation capabilities make older adults more susceptible to emotionally driven and compensatory forms of digital media dependency (Sen et al., 2022). Rather than conducting a direct comparative analysis with younger populations, this study places older adults at the core of investigation, aiming to develop a predictive model tailored to their unique usage patterns and psychosocial conditions.

While conventional statistical approaches such as linear regression and structural equation modeling offer clear advantages in theory testing (Tomarken and Waller, 2005; Nimon, 2012), they may fall short in capturing complex nonlinear relationships or multivariate interaction effects present in real-world data. Moreover, correlation-based studies tend to focus on significance testing, frequently neglecting the marginal contributions and relative importance of different predictors in individual-level risk assessments, which in turn limits their practical value for personalized interventions and risk screening.

In recent years, a growing number of prediction-oriented studies have begun to incorporate machine learning techniques to identify key features associated with internet addiction and problematic digital dependency behaviors (Lee and Kim, 2021; Giraldo-Jiménez et al., 2022; Xiao et al., 2023; Kim K. et al., 2024). These studies, primarily situated within the fields of medicine, health sciences, and information engineering, have demonstrated the significant potential of data-driven approaches in modeling dependence risk.

However, current research still lacks a systematic exploration of how older adults’ usage behaviors, cognitive attitudes, and sociodemographic characteristics jointly contribute to the formation of smartphone overdependence. As such, a critical challenge in this domain lies in balancing theoretical interpretability with the need to move beyond linear assumptions. There is an urgent need to systematically identify and model the complex interplay among multidimensional behavioral features, in order to more accurately capture the mechanisms underlying smartphone overdependence in later life.

To better understand the influencing factors of smartphone overdependence among older adults, this study draws upon the Digital Media Dependency Behavior framework. When digital media become the primary channel for emotional regulation and social interaction, individuals are more likely to develop substitutive and avoidant patterns of technology use, which can eventually lead to psychologically dependent usage (Meshi et al., 2020; Gonçalves et al., 2023; Lv et al., 2025). After retirement, older adults experience a contraction in social roles and external relational networks, making them particularly vulnerable to non-functional smartphone dependence in the domains of emotional regulation, information acquisition, and social maintenance.

Building on this foundation, the study introduces an interpretable machine learning framework to construct a predictive model of smartphone overdependence tailored to the older population. Using the feature importance mechanisms within the XGBoost algorithm, we identify key predictors and quantify their relative contributions to overdependence risk. The study pursues three primary objectives:

To identify critical predictors of smartphone overdependence among older adults.

To assess the relative influence of behavioral, cognitive, and demographic factors on the risk of overdependence.

To develop an interpretable predictive modeling framework that can inform the design of screening tools and intervention strategies for digital health management in later life.

By embedding multidimensional usage behaviors, cognitive attitudes, and demographic characteristics into an interpretable analytical framework, this study advances methodological innovation in modeling smartphone use risks among older adults. The proposed approach offers evidence-based early warning tools to support policymakers and digital literacy practitioners in fostering healthier patterns of technology engagement among aging populations.

## 2 Literature review

### 2.1 Smartphone overdependence

Discussions on problematic smartphone use encompass various terms such as smartphone addiction, smartphone overdependence, and problematic smartphone use, yet a consensus on diagnostic criteria has not been reached. Since 2004, the National Information Agency (NIA) of South Korea has been conducting an annual national survey on internet and smartphone dependency to prevent addiction (National Information Society Agency, 2023). Since 2016, the term “smartphone addiction” has been replaced by “smartphone overdependence.” “Smartphone overdependence” is defined as a state characterized by a significant increase in smartphone use, a decrease in control over its usage, and negative consequences arising from excessive use (Kim S. H. et al., 2024; National Information Society Agency, 2016). Individuals are classified into three categories based on their risk level: no risk, potential risk, and high risk. The potential risk group is characterized by reduced control over smartphone use, leading to interpersonal conflicts or difficulties in performing daily duties (Kim S. H. et al., 2024). The high-risk group, on the other hand, exhibits a complete loss of control over smartphone use, experiences severe interpersonal conflicts, and encounters significant problems in fulfilling daily responsibilities and maintaining health (Kim S. H. et al., 2024). This shift in terminology from “addiction” to “overdependence” reflects a change in government policy, emphasizing individual agency and choice rather than pathologizing smartphone use issues, while also recognizing the essential role digital devices play in modern life.

In recent years, scholars have increasingly adopted multidimensional perspectives to develop a more systematic understanding of digital device dependency. On one hand, digital device dependence has been defined as a persistent and poorly self-regulated pattern of use, characterized by psychological preoccupation, behavioral dysregulation, and functional impairment related to device engagement (Gonçalves et al., 2023). This definition highlights the deep involvement of psychological mechanisms in digital media use. On the other hand, a substantial body of empirical research has confirmed significant associations between individuals’ psychological experiences—such as loneliness and anxiety—and problematic technology use, suggesting that mental health conditions play a crucial role in both the development and maintenance of digital dependence (Elhai et al., 2017; Meshi et al., 2020). When digital media become the primary channel for emotional regulation and social interaction, individuals are more likely to develop substitutive and avoidant patterns of technology use, which can eventually lead to psychologically dependent usage (Meshi et al., 2020; Gonçalves et al., 2023; Lv et al., 2025). Further studies have pointed out that digital vulnerability—including limited digital skills, impaired emotional regulation, and lack of social support—can accumulate over prolonged periods of media use and, in the absence of effective coping mechanisms or interventions, may evolve into more stable patterns of dependent behavior (Livingstone and Helsper, 2007; Lv et al., 2025). Therefore, smartphone overdependence should be understood as the outcome of prolonged and multifaceted mechanisms rather than a simple reaction to any single variable. It is essential to identify the underlying behavioral logics, cognitive tendencies, and demographic characteristics that together form complex pathways leading to dependence.

User behavior characteristics (such as preferences for the use of smart devices) reflect an individual’s psychological attachment to the functions of different media (Elhai et al., 2017; Panova and Carbonell, 2018; Jeong and Bae, 2022). These behaviors are not merely quantitative indicators; rather, they offer valuable insights into the underlying psychological needs and motivational types that drive usage. For example, research has shown that compared to informational use, entertainment-oriented activities (e.g., gaming, video streaming, and social media engagement) are more likely to elicit instant gratification, thereby increasing the propensity for dependency (Jeong and Bae, 2022). Cognitive and attitudinal features (such as one’s subjective awareness of overuse and receptiveness to healthy usage strategies) constitute the psychological mechanisms through which digital vulnerability may evolve into behavioral dependence (Meshi et al., 2020). These variables indicate whether individuals possess self-awareness, self-regulation, and reflective capacities regarding their device use, serving as critical signals for identifying users at high risk of overdependence (Kim S. H. et al., 2024). In addition, demographic characteristics—such as age, educational attainment, and income level—play a significant moderating role in the developmental trajectory of digital dependence (Hunsaker and Hargittai, 2018; Beneito-Montagut et al., 2022). These factors represent individuals’ life-course attributes and shape how they engage with digital technologies in terms of access, intensity, and meaning. As such, they provide essential contextual conditions for understanding digital media usage patterns and pathways of risk exposure.

Therefore, the formation of smartphone overdependence behavior is characterized by distinct multidimensional and interactive features. The associated risk pathways encompass both micro-level psychological and behavioral factors, as well as macro-level structural variables embedded within broader social contexts. To accurately predict smartphone overdependence and identify its key influencing factors, it is necessary to incorporate the interactive logic of these multidimensional mechanisms into the modeling process. Compared to traditional regression-based approaches, machine learning methods are better equipped to capture nonlinear relationships and complex interaction effects among variables. As such, they offer a powerful tool for developing predictive models of smartphone overdependence among older adults. Guided by this rationale, the present study constructs a multi-source variable prediction model of digital overdependence behavior in later life. The model aims to identify the most salient predictors of smartphone overdependence among older adults, thereby providing both empirical evidence and theoretical grounding for the design of targeted intervention strategies.

### 2.2 Application of machine learning to smartphone overdependence research

As an important branch of artificial intelligence, machine learning is committed to imitating the human learning process through the combination of mathematics, statistics, cognitive science and computer science. By analyzing historical data to identify patterns, it can predict future trends (Al-Hashem et al., 2021; Ahmadi et al., 2023). Its applications have profoundly impacted various fields, including disease diagnosis (Aruleba et al., 2022), fraud detection (Khan et al., 2022), text classification (Chen et al., 2022), and image recognition (Graham et al., 2022).

In recent years, there have also been studies utilizing machine learning techniques to explore the phenomenon of smartphone overdependence. For example, Kim K. et al. (2024) employed machine learning algorithms to predict high-risk groups for smartphone addiction using data from the past 5 years, achieving an accuracy rate of 87.60%. Xiao et al. (2023) used several different machine learning algorithms to predict problematic smartphone use, finding that shrinkage algorithms (lasso, ridge, and elastic net regression) outperformed others, and identified FoMO (fear of missing out), emotional, and cognitive self-regulation as key features for predicting problematic smartphone use. Giraldo-Jiménez et al. (2022) developed a data-driven smartphone dependency prediction model using machine learning techniques, and through stratified k-fold cross-validation, found that classifiers such as random forests, logistic regression, and support vector machines had the highest prediction accuracy for smartphone dependency, reaching 76–77%. They also noted that self-reported information helps accurately predict smartphone overdependence. Additionally, Ellis et al. (2019) used the k-means clustering algorithm to cluster users with similar smartphone usage behaviors and analyzed users’ smartphone usage behaviors using objective behavioral data retrieved from “Screen Time” application. Lawanont and Inoue (2017) developed a system to identify smartphone addiction by leveraging usage data. Their approach included implementing classification models such as Naive Bayes, Decision Trees, K-Nearest Neighbors, and Support Vector Machines to assess the probability of smartphone addiction. Additionally, Kim and Lee (2020) used Decision Tree, Random Forest, and Support Vector Machine techniques to diagnose and predict smartphone overdependence, finding that variables influencing the classification rate of smartphone overdependence included types of life services, information retrieval, and leisure pursuits. Through decision tree analysis, they identified that the most significant influencing factor for smartphone overdependence was the user’s actual perception of overdependence. In summary, machine learning algorithms hold significant value in predicting and understanding smartphone overdependence, particularly in the social sciences.

The advantages of machine learning lie in its ability to assess both linear and nonlinear relationships among variables and to generate models through classification and numerical prediction variables. In traditional statistical methods such as regression analysis, when multiple variables are used simultaneously, fundamental assumptions about the exogeneity and homoscedasticity of the independent variables become difficult to maintain, and high correlations among variables can lead to multicollinearity issues (Woo, 2022). However, machine learning-based predictive models can analyze the relationships between dependent and independent variables, and their predictive capabilities remain unaffected even in the presence of multicollinearity (Varian, 2014). This study selected data from a South Korean terminal data survey, which included approximately 140 questions covering participants’ demographic characteristics, smartphone usage status, awareness of overdependence issues, and psychosocial characteristics as independent variables for analyzing the risk factors of smartphone overdependence. To determine the importance of variables related to smartphone overdependence, we employed the XGBoost algorithm to comprehensively analyze the feature factors that might influence smartphone dependence. The machine learning algorithms utilized in this study encompass a range of representative classification techniques, including Logistic Regression (LR), Decision Tree (DT), Naïve Bayes (NB), Support Vector Machine (SVM), K-Nearest Neighbors (KNN), and XGBoost (XGB). We aim to identify the most accurate classification analysis methods and the key variables that impact classification accuracy.

**LR** is a widely used statistical method and machine learning algorithm for binary classification problems that uses logistic or sigmoid functions to model the probability of events (Islam et al., 2023). LR shows the relationship between features and calculates the probability of a particular outcome. It is widely used in predictive modeling due to its relative accuracy, simplicity of construction process, low likelihood of overfitting, and excellence in finding relationships that minimize error (Friedman et al., 2000; Lever et al., 2016). Its specific formula is as follows:


$$h_{\Theta }(x)=\frac{1}{1+e^{-(\beta_{0}+\beta_{1}X)}}$$

Where $h_{\Theta }(x)$ is the output of logistic function, which ranges from 0$\leq h_{\Theta }(x)\leq 1$. $\beta_{0}\;$is the y-intercept. $\beta_{1}$is the slope. X is the independent variable.

**Figure 1:**
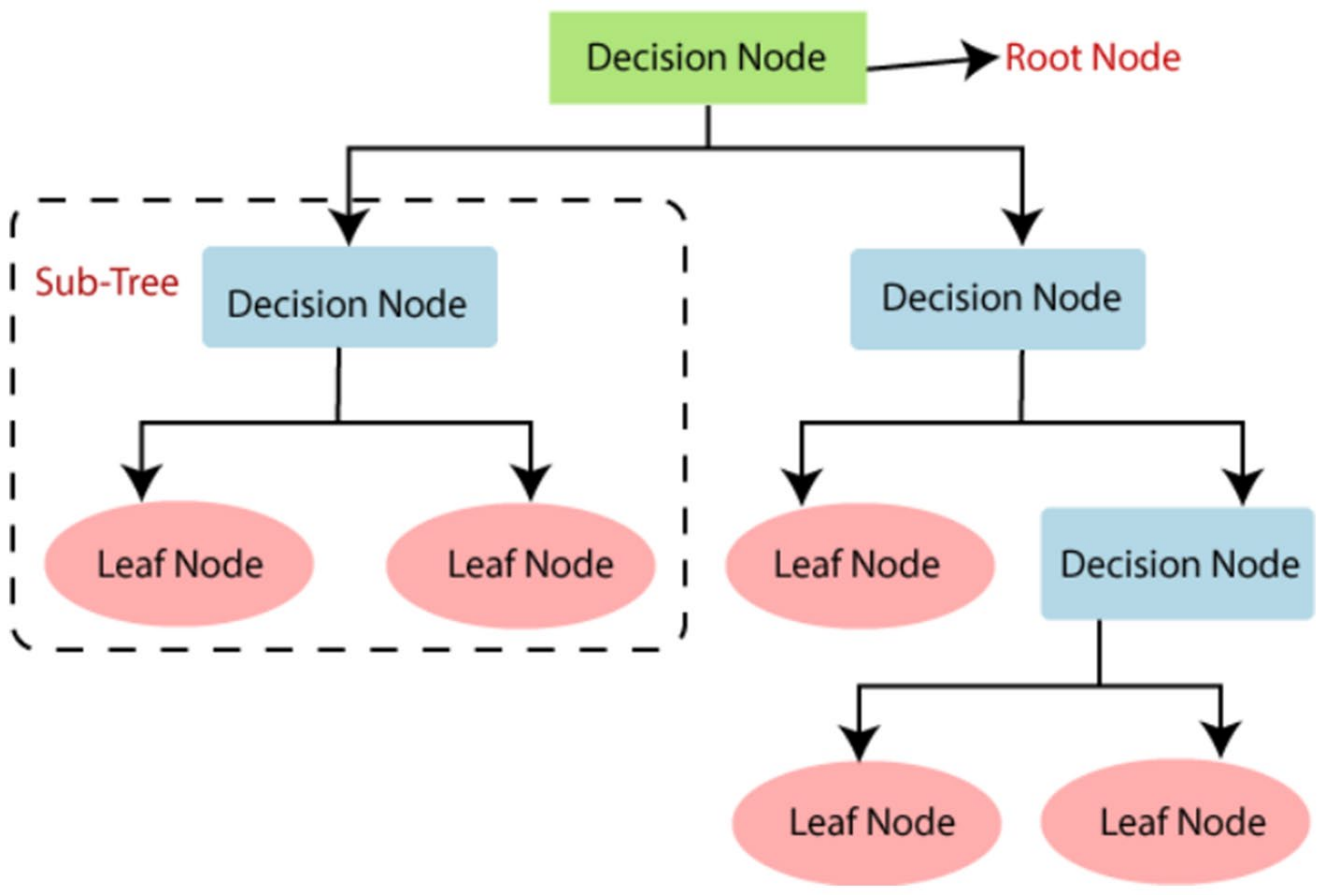
Decision tree model.

**DT** is one of the most useful methods for classification and predictive analytics in supervised learning. The inference rules are unfolded like a tree, making the decision-making process and results visually clear (Charbuty and Abdulazeez, 2021). DT performs classification and prediction through four stages: in the first stage, the decision tree is generated. Appropriate splitting criteria and stopping rules are determined based on the analysis objectives. In the second stage, pruning is performed. Branches that may significantly increase classification errors or have inappropriate inference rules are removed. In the third phase, validity assessment is performed. Cross-validation is analyzed based on benefit maps, risk maps, or validation data. In the final stage, interpretation and prediction are performed. The entire decision tree is interpreted and predictive modeling is done. DT can be interpreted in various forms depending on how the splitting criteria, stopping rules, and evaluation criteria are applied. Compared to other methods, DT is usually simpler in data preparation and can handle a wide range of data types, enabling it to generate valid results for important data sources in a relatively short time (Lee et al., 2022). The conceptual model is shown in Figure 1.

**NB Classifier** is a theory-based supervised learning model for data classification. It is a conditional probability-based classification method that classifies data by calculating the probability that the features belong to each category (Miljković et al., 2010). The method classifies each data by calculating the probability that an input feature belongs to a specific category relative to the overall category probability distribution through Bayes’ theorem. The formula for Bayes’ theorem is:


$$P(A|B)=\frac{P(B|A).P(A)}{P(B)}$$

Where *P(A│B)* is the probability of event *A* occurring conditional on event *B* occurring (a posteriori probability). *P(B│A)* is the probability (likelihood probability) of event *B* occurring conditional on event *A* occurring. *P(A)* is the probability of event *A* occurring (prior probability). *P(B)* is the probability of event *B* occurring (marginal probability).

**Figure 2:**
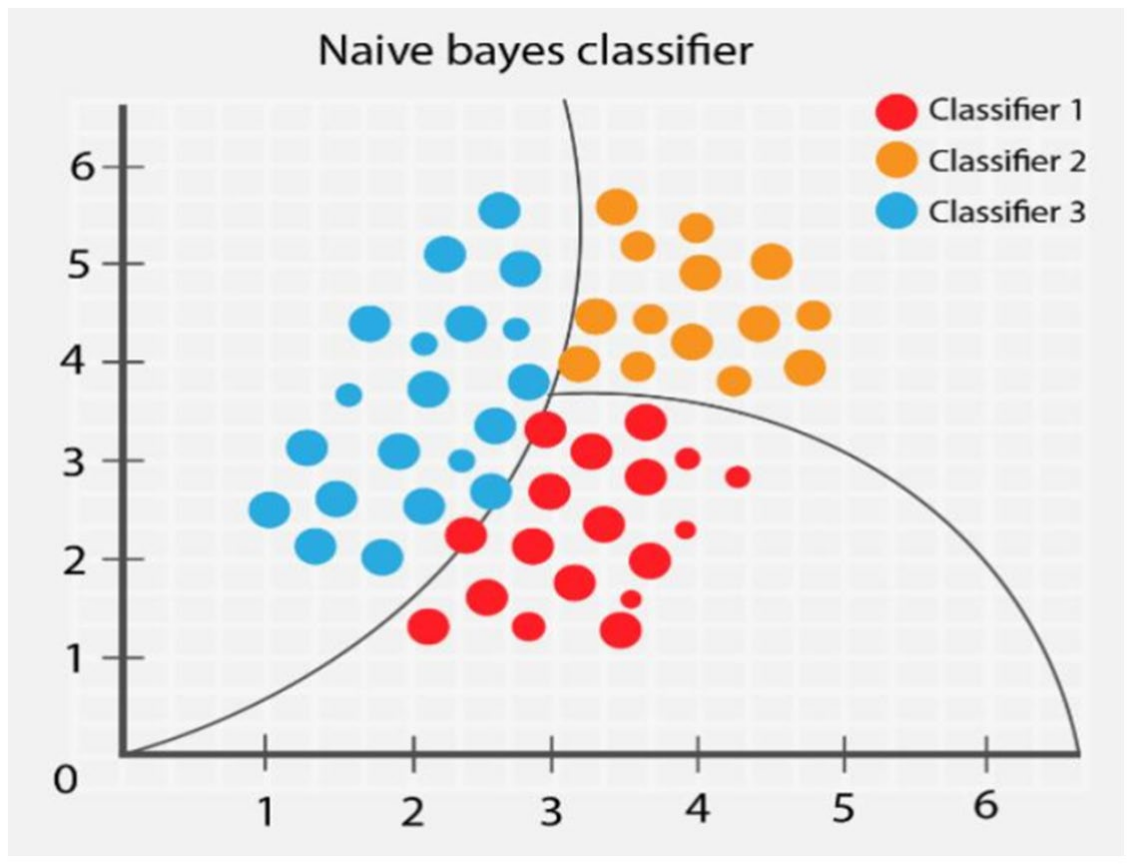
Naïve Bayes model.

The NB classifier estimates the posterior probability of each class label by calculating the eigenvalues and assigns instances to the class with the highest posterior probability. The probabilistic nature of the algorithm allows it to measure the likelihood of different outcomes based on the input data and thus make reliable decisions in the prediction process. NB has the advantages of simplicity, speed, and efficiency, and has been successfully applied in the areas of text classification, spam detection, and sentiment analysis (Carvalho and Plastino, 2021; Omotehinwa and Oyewola, 2023). Its conceptual diagram is shown in Figure 2.

**Figure 3:**
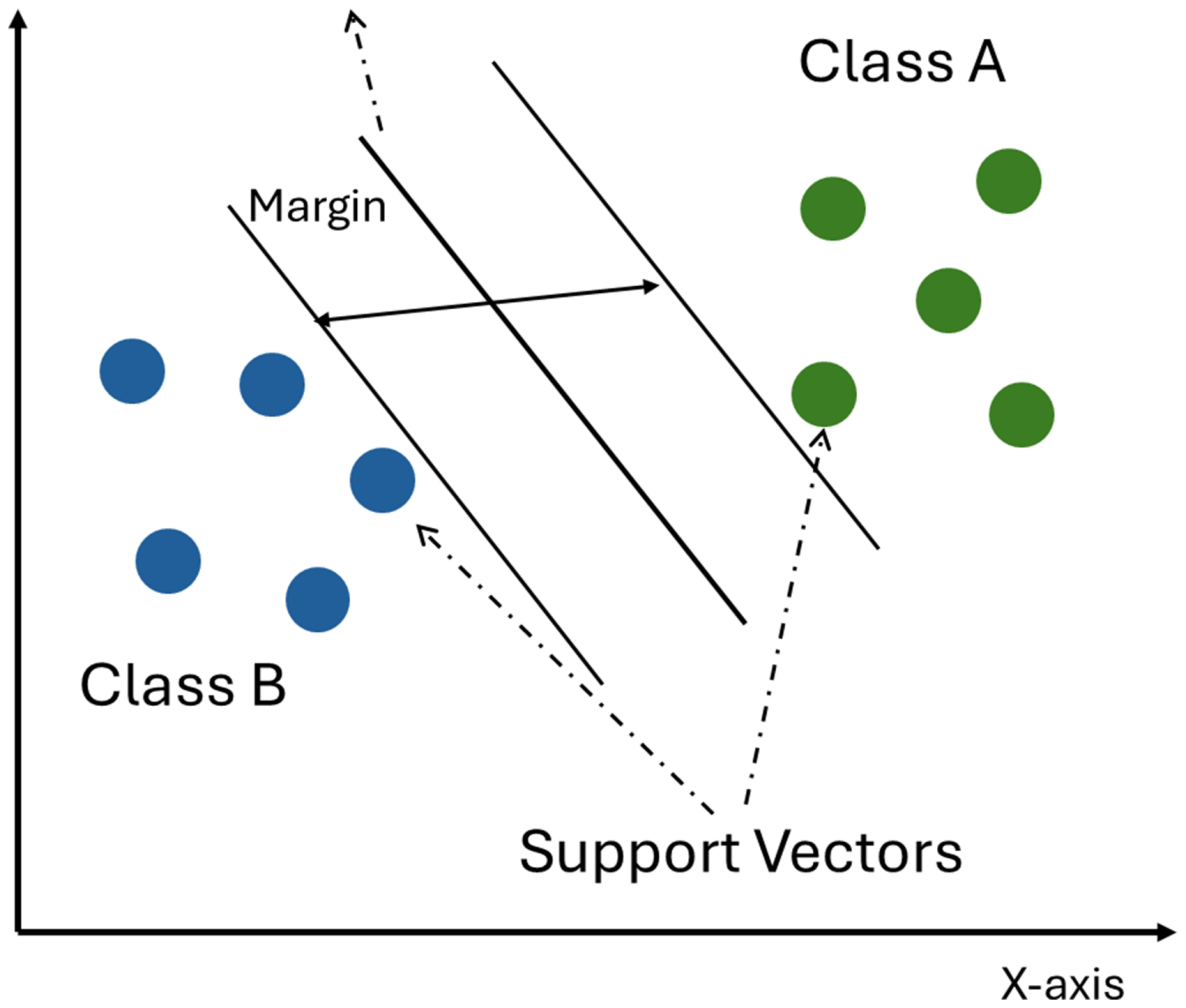
SVM model.

**SVM** is a classification technique that tends to accurately classify data from various distributions. Due to its ability to efficiently and accurately deal with linear and nonlinear problems, SVM has been successfully used in various fields such as bioinformatics, finance, and image recognition. SVM model separates the data by finding the optimal boundary in the three-dimensional space, which is called hyperplane (Pisner and Schnyer, 2020). As shown in Figure 3, the least misclassification of new data occurs when the hyperplane with maximum spacing is used as a classifier. SVM is a classification method that finds the Maximum Margin Hyperplane (Srinivasarao and Sharaff, 2023). Its expression is as follows:


$$f(x)=\omega^{T}\emptyset (x)+b$$

The equation aims to find a function *f(x)* whose weight vector $\omega^{T}\emptyset (x)\;$is within the smallest possible range of deviations from the actual target value of a given input vector *x*, where the superscript denotes the transpose of the matrix and *b* is the bias.

**Figure 4:**
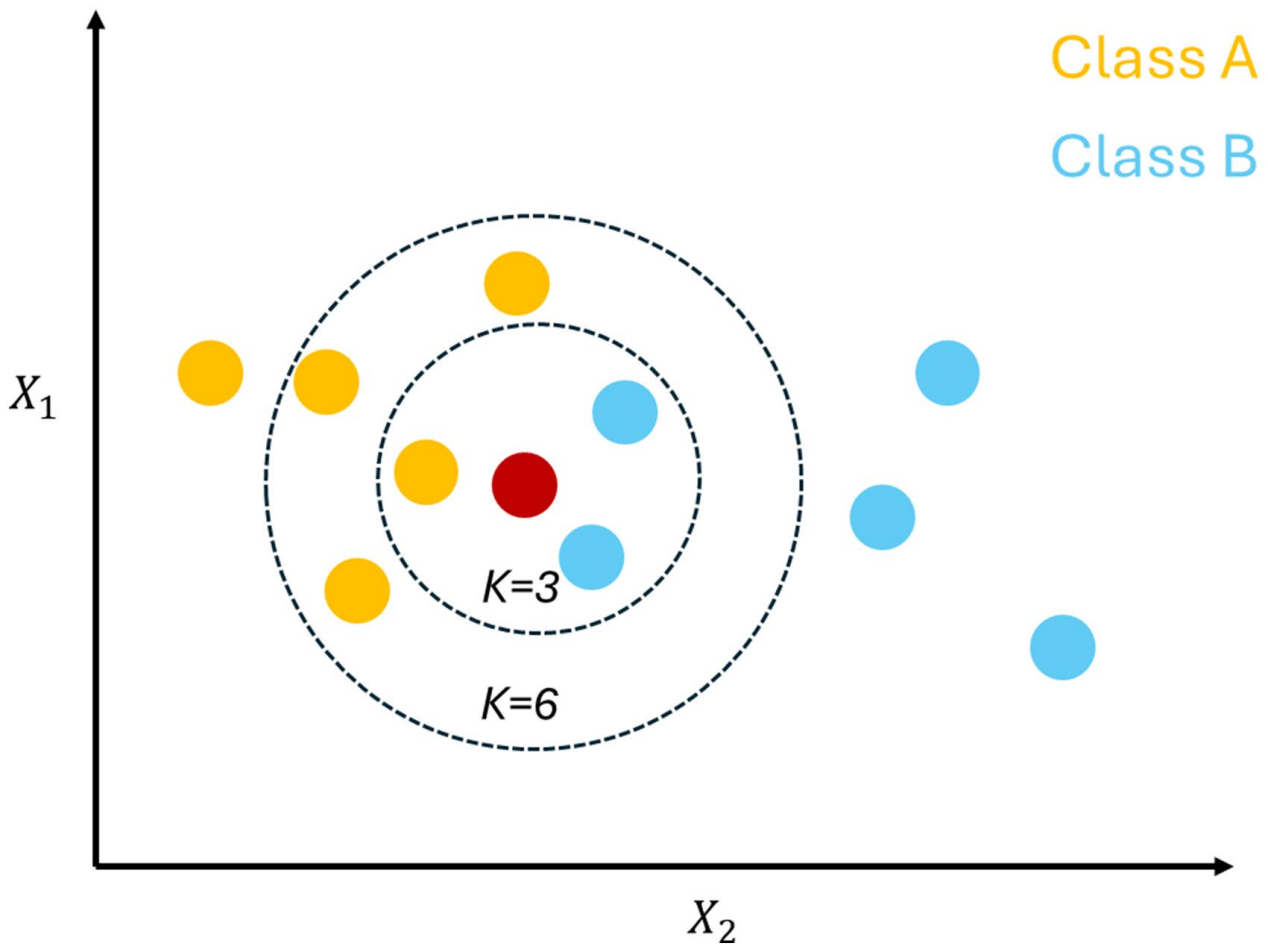
KNN model.

**KNN** is a theoretically sophisticated and simple machine learning algorithm. The basic idea is that in the feature space, if most of the samples in the nearest k neighbors of a given sample belong to a particular category, the sample also belongs to that category (Liu et al., 2019). The conceptual model of the KNN algorithm is shown in Figure 4.

In Figure 4, K-NNN works by input parameter *k*. The figure shows that there may be two or more categories and *k* indicates the number of nearest neighbors. At this point, the criterion for determining the nearest neighbors is to use the Euclidean distance metric:


$$d=\sqrt{{\left(x_{1}-x_{2}\right)}^{2}+{\left(y_{1}-y_{2}\right)}^{2}}$$

When *k* is 3, the three nearest neighbors can be selected to arrive at a preference by using the above formula. In the case of Figure 4, when *k* is 3, the result favors category *B*, but if k is changed to 6, the result may favor category *A*. The algorithm, in addition to preferences, can predict the future based on current data, making it suitable for a wide range of applications.

**Figure 5:**
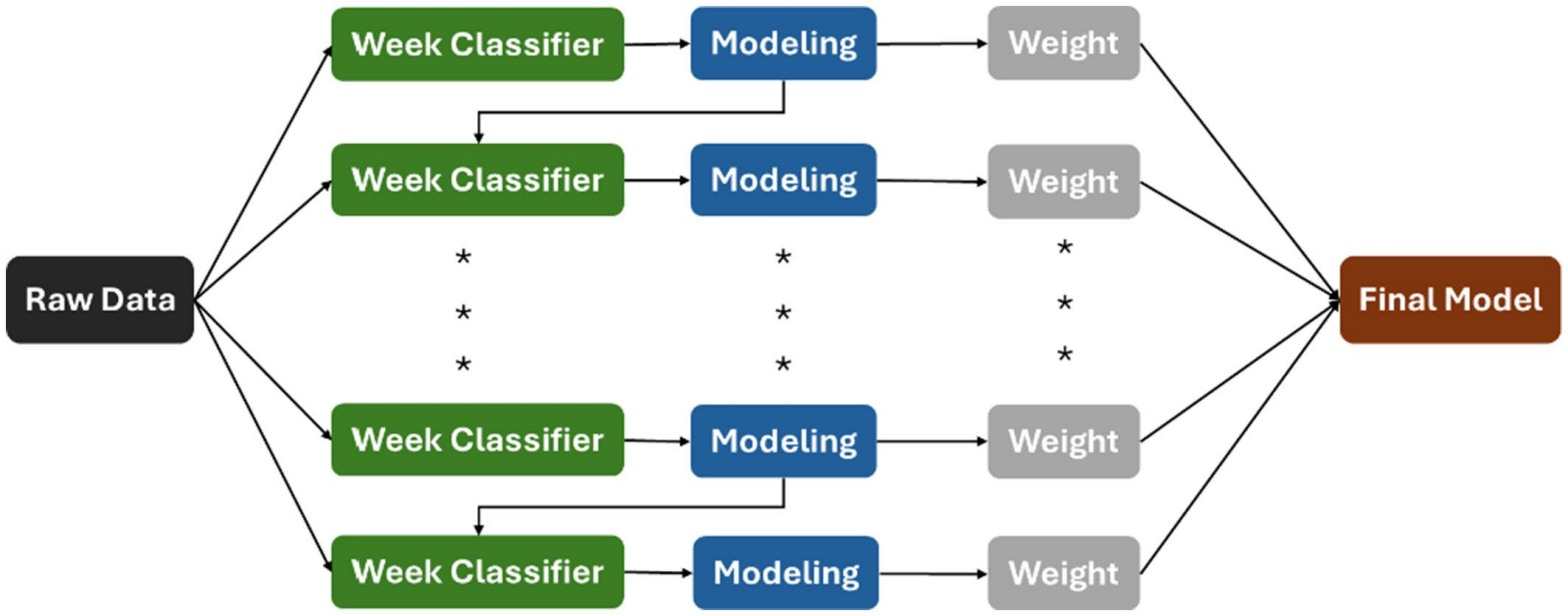
XGB model.

**XGB** technique is an integrated technique for generating learners with strong predictive power by combining learners with weak predictive power (Weak learner; Atef et al., 2022). The core principle of XGBoost is to configure an iterative process of a powerful learner by optimizing an objective function consisting of a loss function and a regularization term. The loss function measures the difference between the predicted and actual results, while the regularization term punishes complex models to prevent overfitting (Su et al., 2023). XGBoost is less demanding and more effective in processing categorical or continuous features in the data, and with numerous advantages including efficiency, scalability, and the ability to deal with missing values, XGBoost can be an effective solution to a various prediction task. The conceptual diagram of XGBoost is shown in Figure 5.

## 3 Materials and methods

### 3.1 Participants and data preprocessing

This study employed panel data from the 2023 Smartphone Overdependence Survey conducted by the Korea Information Society Agency (National Information Society Agency, 2023), based on structured questionnaire responses. Data collection took place from August to October 2023, targeting smartphone (internet) users aged 3 to 69 across 10,000 households nationwide. The data possesses national representativeness and statistical generalizability, as they are based on annual uniform surveys conducted with randomly selected households from across the country. This anonymized dataset was accessed from the public data portal of South Korea,^1^ and data on older adults were selected. All variables included in this study were derived from this extracted dataset.

This study adopts a quantitative approach to predict the risk of smartphone overdependence, with particular attention to the recent surge in smartphone usage among older adults in South Korea. Given the increasing prevalence of this phenomenon, the risk of overdependence in this demographic warrants close examination. To ensure the relevance of participants’ usage experiences, inclusion criteria required individuals to have used a smartphone within the past month. Those who had not used a smartphone for more than 1 month or who were unable to use one due to illness or medical conditions were excluded from the sample. During structured interviews, each participant answered approximately 150 to 180 questions, which were systematically organized and analyzed across seven thematic categories. The survey items were based on the 2023 National Information Society Agency (NIA) questionnaire and were informed by previous studies, including Park et al. (2021), Bertocchi et al. (2022), Jeong and Bae (2022), Li et al. (2022), Ren and Klausen (2024), Kim S. H. et al. (2024), and Wang et al. (2024). These sources guided the naming and categorization of variables used in the present study (see Table 1). The sampling followed a square root proportional allocation method, based on key sociodemographic variables—such as gender, age, and geographic location—collected via multistage stratified sampling. As a result, the dataset is well-suited for the current analysis, comprising responses from 4,338 individuals aged 60 and above. Table 2 presents the descriptive statistics and demographic characteristics of the sample.

**Table 1:** Contents of the smartphone overdependence survey.

Category	Contents	Indicators
Demographic and socioeconomic characteristics	Age, gender, educational attainment, occupation, monthly household income, size of residential area (metropolitan/small-medium city/rural), type of housing (apartment, detached house, rental housing, etc.)	Dummy, Categorical
Digital access conditions	Ownership and usage of smartphones; Internet access via smartphone	Dummy
Smartphone usage patterns	Types of usage (e.g., social communication, entertainment, life services, information seeking and learning, economic transactions, work-related tools); Time allocation across usage types; Self-perceived control over usage time and compulsiveness across usage categories	4-point Likert scale (Strongly Disagree → Strongly Agree)
Online video service usage	Frequency of use; Types of content viewed (entertainment, informational, violent, sexual, gambling, etc.); Platform preference (e.g., YouTube, TikTok); Proportion of time spent; Perceived control over video usage (e.g., difficulty controlling or addiction to short video content)	Categorical; 4-point Likert scale (Strongly Disagree → Strongly Agree)
Use of smartphone overdependence prevention and intervention services	Participation in preventive education programs; Awareness and experience with counseling or intervention services; Subjective evaluation of effectiveness; Willingness to participate in or recommend future services	Dummy; 4-point Likert scale (Strongly Disagree → Strongly Agree)
Awareness and coping strategies related to smartphone overdependence	Awareness of causes, coping strategies, barriers, and efforts to reduce overdependence; Subjective assessment of the severity of the problem (awareness, attitudes, and suggested preventive actions); Perceptions of responsibility among different social actors (e.g., individuals, families, corporations, government, educational institutions); Understanding of preventive and intervention measures; Self-management behaviors in daily life	Categorical; 4-point Likert scale (Strongly Disagree → Strongly Agree)
Psychosocial characteristics	Digital literacy skills (ability to access, evaluate, produce, and apply information in digital contexts); Social support and trust networks (perceived interpersonal support and perceived fairness in society); Life satisfaction and subjective well-being (satisfaction across various life domains)	4-point Likert scale (Strongly Disagree → Strongly Agree)

**Table 2:** Characteristics of demographic.

Classification		N	%
Gender	Male	2,141	49.4
	Female	2,197	50.6
Age	60–64	2,246	51.8
	More 65	2092	48.2
Education	Less than primary education	146	3.4
	middle school graduate	693	16.0
	High school graduate	3,065	70.7
	Bachelor’s degree	422	9.7
	Graduate degree	7	0.2
	No response	5	0.1
Monthly house income (KRW)	below 2,000,000	639	14.7
	2,000,000–3,990,000	1963	45.3
	4,000,000–5,990,000	1,006	23.2
	6,000,000–7,990,000	528	12.2
	More 8,000,000	202	4.7
Occupation	Managers	41	0.9
	Professionals and related workers	47	1.1
	Clerical workers	105	2.4
	Service workers	742	17.1
	Sales workers	606	14.0
	Skilled agricultural, forestry and fishery workers	319	7.4
	Craft and related trades workers	474	10.9
	Plant and machine operators and assemblers	194	4.5
	Elementary occupations	690	15.9
	Full-time homemaker	849	19.6
	Unemployed	260	6.0
	Other	11	0.3
City size	Large city	1808	41.7
	Medium/Small city	1755	40.5
	Township/Rural area	775	17.9
House	Detached house	1,381	31.8
	Apartment	2,129	49.1
	Row house / multi-family housing	713	16.4
	Other (officetel, residence within commercial building, etc.)	115	2.7
Total		4,338	100

### 3.2 Predictor variables

In this study, we use a binary dependent variable to predict the factors influencing smartphone overdependence among older adults. As shown in Table 3, smartphone overdependence was measured using 10 items. These items assessed the difficulty in controlling smartphone usage, the frequency of attention disruption caused by smartphone use, and the occurrence of health, occupational, or interpersonal conflicts due to smartphone usage (Kim S. H. et al., 2024; Jeong and Bae, 2022). All items were rated on a 4-point scale. According to the 2023 Smartphone Overdependence Survey Report, individuals scoring 28 or above on the 10-item scale are classified as high-risk, while those scoring between 24 and 27 are classified as potential risk (National Information Society Agency, 2023). This study sets the threshold for smartphone overdependence at 24 points or above, coding these users as 1, with others coded as 0. This standard is based on the comprehensive smartphone dependence scale created by the Korea Information Society Development Institute in 2011 (Kim S. H. et al., 2024; Lee et al., 2023). Among the 4,338 participants examined, 566 (13.05%) older adults were identified as high-risk for smartphone overdependence, while 3,772 (86.95%) older adults were identified as low-risk.

**Table 3:** Predictor variable.

Measurement items	
Self-control failure	(1) I fail every time I try to reduce my smartphone usage time.
	(2) It is difficult to control the amount of time I use my smartphone.
	(3) It is challenging to maintain an appropriate amount of smartphone usage time.
Salience	(4) It is hard to concentrate on other tasks when the smartphone is nearby.
	(5) Thoughts of the smartphone constantly occupy my mind.
	(6) I strongly feel the urge to use my smartphone.
	(7) I have experienced health problems due to smartphone usage.
Problematic consequences	(8) I have had severe arguments with my family because of my smartphone usage.
	(9) I have experienced severe conflicts in social relationships due to my smartphone usage.
	(10) I have difficulty performing my work because of my smartphone usage.

### 3.3 Explanatory variables

**Figure 6:**
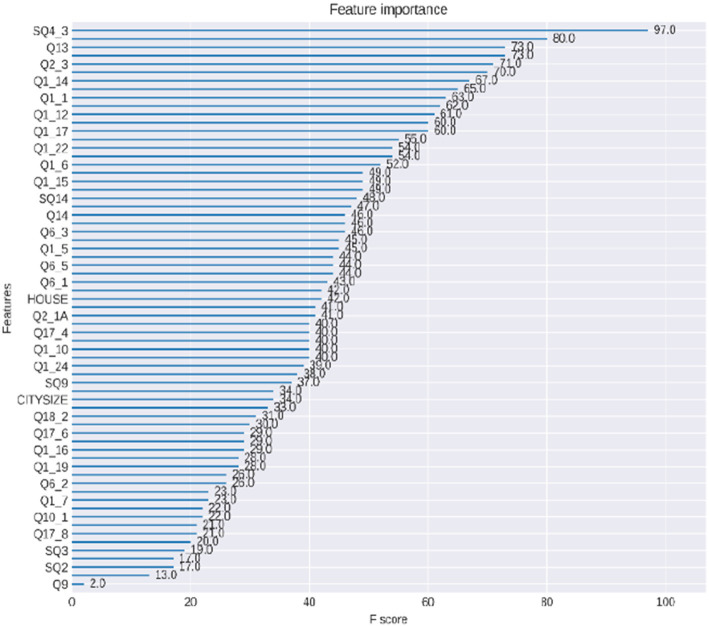
Feature importance.

We used XGBoost’s feature importance algorithm to identify the most critical explanatory variables in the predictive model. The feature importance algorithm improves the performance of the classifier by eliminating redundant and irrelevant features (Jiang et al., 2022). Initially, we excluded variables related to personal identity information and entries with missing data from the original dataset of 4,338 records, which left us with 80 explanatory variables. We then applied the XGBoost algorithm to compute the importance scores of these 80 variables, identifying the key factors that most significantly influence the predictive outcomes. The importance scores of all features are illustrated in Figure 6. Given that the data structure for smartphone overdependence among older adults lacks uniformity in dimensions and units, this may impact the model’s assessment of feature weights, thus affecting its accuracy and convergence speed. Therefore, we used the Min-Max Scaler method to normalize the data to values between 0 and 1. From the 80 explanatory variables, we selected the top 15 most important variables based on their importance scores, forming the optimal feature subset. The results of the feature selection are detailed in Table 4.

**Table 4:** Explanatory variables.

No.	Variables	Measurement items
1	SQ4_3	Age
2	DQ4	Occupation
3	Q13	Awareness of Solutions for Overdependence Issues _ Measures to Actively Address Smartphone Overdependence and Promote Healthy Usage
4	Q2_5	Proportion of Smartphone Usage Time_ Information Search and Education
5	Q2_3	Proportion of Smartphone Usage Time_ Leisure Activities
6	Q1_3	Smartphone usage content _Messenger
7	Q1_14	Smartphone usage content _Music
8	Q5	Awareness of Personal Smartphone Overdependence
9	Q1_1	Smartphone usage content _Social Networking Services (SNS)
10	Q2_1	Proportion of Smartphone Usage Time_ Communication
11	Q1_12	Smartphone usage content _Games
12	Q1_23	Smartphone usage content _Hobby Search
13	Q1_17	Smartphone usage content _Photography (Shooting and Editing) and Drawing
14	Q1_21	Smartphone usage content _News Viewing
15	Q1_22	Smartphone usage content _Academic/Work-Related Search

### 3.4 Feature correlation test

This study utilizes the Pearson correlation coefficient to analyze the relationship between different features and constructs a correlation matrix to assess the level of correlation between these selected features. The Pearson correlation coefficient (*Cc*) is a widely used method for measuring feature correlation. It calculates the correlation between variables *X* and *Y*, with the result denoted as *r(X, Y).*


$$r(X,Y)=\frac{\sum (x_{i}-{\bar{x}}_{i})(y_{i}-{\bar{y}}_{i})}{\sqrt{\sum_{i}{\left(x_{i}-{\bar{x}}_{i}\right)}^{2}}\sqrt{\sum_{i}{\left(y_{i}-{\bar{y}}_{i}\right)}^{2}}}$$

The correlation coefficient (*r*) measures the strength and direction of the relationship between two variables, *X* and *Y*. When *X* and *Y* are independent, the correlation coefficient is zero, as there is no predictable relationship between the two variables.

In a dataset containing m samples and n features, the Pearson correlation coefficient can be calculated between every pair of features to create a correlation matrix, *R*(*p_ij*). This matrix displays the correlation between each feature *i* and *j*. By analyzing these correlations, insights into the relationships between different features can be gained, providing valuable information for predictive modeling and data analysis.


$$\left[\begin{array}{cccc} p_{11} & p_{12} & \ldots & p_{1n}\\ p_{21} & p_{22} & \ldots & p_{2n}\\ \vdots & \vdots & \vdots & \vdots \\ p_{n1} & p_{n2} & \ldots & p_{\mathrm{nn}} \end{array}\right]$$

**Figure 7:**
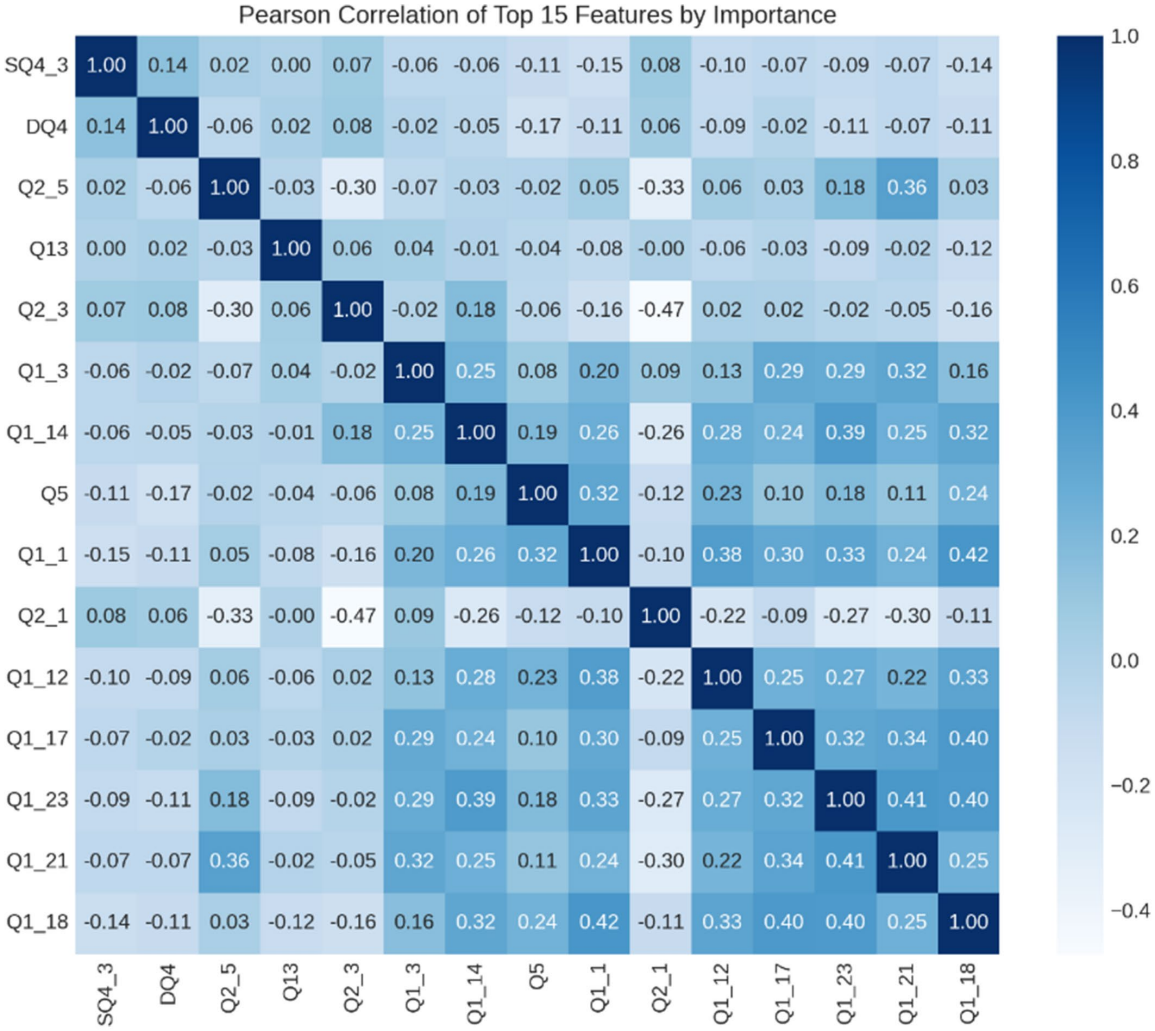
Feature correlation heatmap.

Figure 7 illustrates a heat map of the calculated feature correlations. The results show that the correlations among the 15 feature vectors are relatively weak. The lowest correlation coefficient of 0 is between Q13 and SQ4_3, while the highest correlation coefficient of 0.42 is between Q1_18 and Q1_1. Therefore, the selected features are not redundant.

### 3.5 Evaluation metrics

The evaluation metrics are crucial for assessing the final results. To evaluate the effectiveness of the predictive algorithm for smartphone overdependence among older adults, we derived evaluation metrics such as accuracy, precision, recall, and F1 scores from the confusion matrix, the result is critical. The prediction performance for smartphone overdependence depends on the number of true positives (TP), false negatives (FN), true negatives (TN), and false positives (FP). The calculation of these five indicators needs to be derived using the following formula:

Accuracy:


$$\text{Accuracy}=\frac{\mathrm{TP}+\mathrm{TN}}{\mathrm{TP}+\mathrm{TN}+\mathrm{FP}+\mathrm{FN}}$$

Precision:


$$\text{Precision}=\frac{\mathrm{TP}}{\mathrm{TP}+\mathrm{FP}}$$

Recall:


$$\text{Recall}=\frac{\mathrm{TP}}{\mathrm{TP}+\mathrm{FN}}$$

F1 Score:


$$F1=2\times \frac{\text{Precision}\times \text{Recall}}{\text{Precision}+\text{Recall}}$$

**Figure 8:**
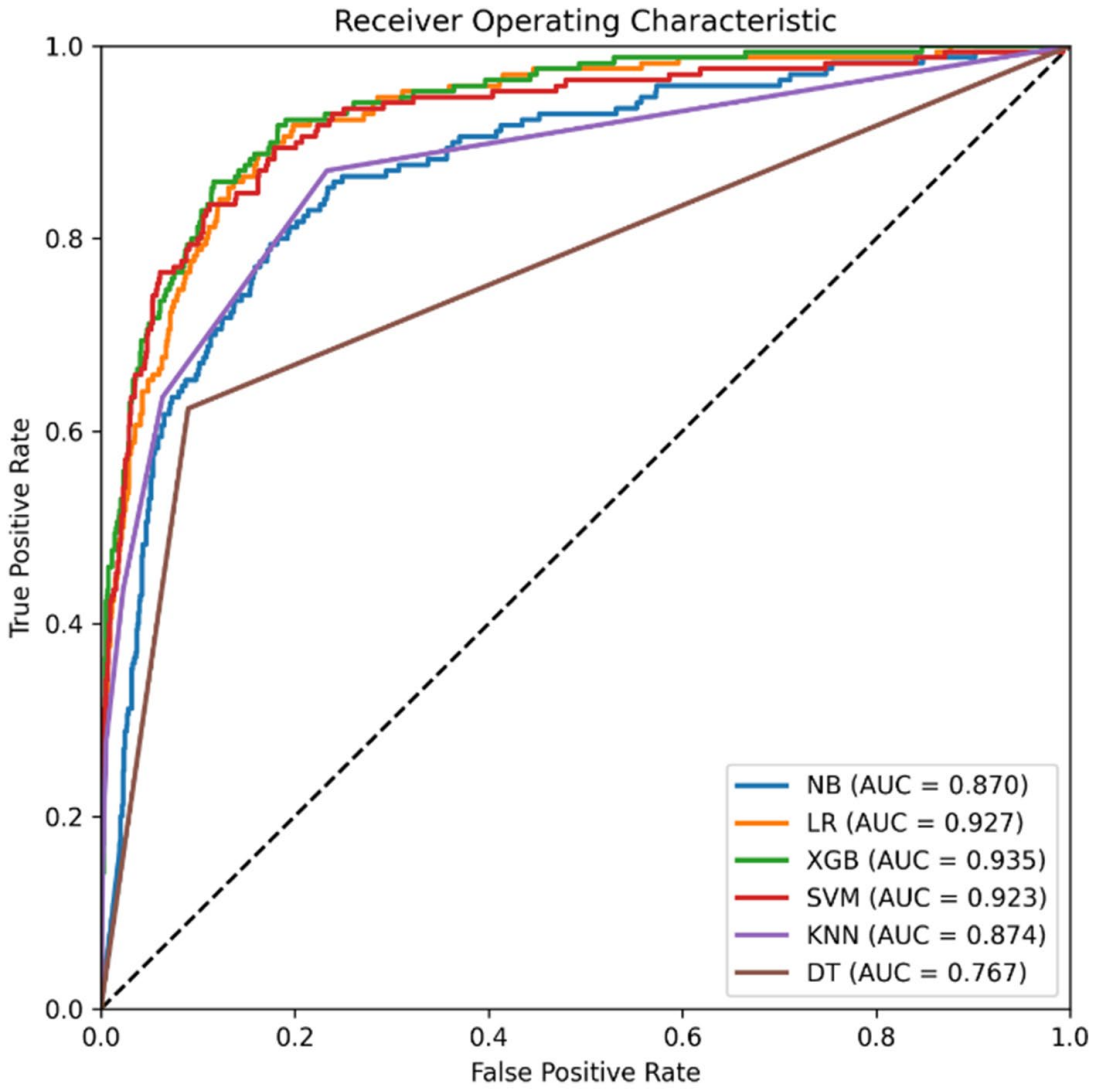
Comparison of ROC curves of models.

We analyzed the receiver operating characteristic curve (ROC) of the confusion matrix and determined the area under the curve (AUC) to assess the effectiveness of the model in predicting smartphone overdependence. Higher values of the AUC indicate better performance and are illustrated in Figure 8. The results show that when these metrics reach significant values, the model’s predictions are considered successful. More details on the performance metrics of the various algorithms can be found in Table 5 for comparison.

**Table 5:** Prediction model analysis results.

Classifier	Accuracy	Precision	Recall	F1 score	AUC
LR	0.914	0.696	0.606	0.648	0.927
DT	0.873	0.510	0.624	0.561	0.767
NB	0.833	0.422	0.753	0.541	0.870
SVM	0.921	0.756	0.582	0.658	0.923
KNN	0.906	0.740	0.435	0.548	0.874
XGBoost	0.925	0.748	0.647	0.694	0.935

This study mainly focuses on evaluating the prediction accuracy of various machine learning based binary classifiers, the main metrics of which are shown in Table 5 and Figure 8. The results show that the prediction accuracies of all these classifiers are high, highlighting their usefulness. Among them, the XGBoost classifier has the highest accuracy of 0.925, which exceeds SVM (0.921), LR (0.914), KNN (0.906), DT (0.873) and NB (0.833). Consequently, the XGBoost classifier emerged as the top choice for predicting smartphone overdependence in older adults. The findings not only validate the superior performance of the XGBoost classifier but also offer valuable insights for future research and practical applications concerning smartphone overdependence in older adults. This study emphasizes the importance of utilizing advanced machine learning techniques like XGBoost in predictive modeling to enhance accuracy and efficiency in addressing behavioral issues such as smartphone overuse.

## 4 Discussion

This study provides insights into the alarming trend of smartphone overdependence among older Koreans by analyzing smartphone usage patterns. By conducting feature importance analysis, four key dimensions were identified that play an important role in predicting the risk of smartphone overdependence among older adults. These dimensions include demographic characteristics, composition of smartphone usage time, awareness of smartphone overdependence issues, and smartphone usage content. These dimensions are substantively significant in understanding and assessing the risk of smartphone overdependence in older adults. According to previous studies, smartphone overdependence requires a comprehensive consideration of sociodemographic characteristics, usage time, and usage type (Jeong and Bae, 2022). As smartphones become more prevalent among middle-aged and older populations, the risks associated with their overuse and dependence become increasingly evident.

This study aimed to delve into the issue of smartphone overdependence among older adults by utilizing cutting-edge machine learning techniques to develop a predictive model. Through this innovative approach, 15 crucial variables were pinpointed and analyzed to gain insights into the risk factors associated with smartphone overdependence. In this process, 15 important predictive variables were identified, covering various aspects, including demographic characteristics (e.g., age, occupation), the composition of smartphone usage time (e.g., the proportion of time spent on communication, leisure, information search, and education), as well as awareness of smartphone overdependence issues (including self-awareness of overdependence and coping strategies), smartphone usage content (e.g., communication content: Messenger, SNS; entertainment content: music, games, photos (shooting and editing), and paintings; information content: interest (hobby) search, news viewing, academic/work search). By identifying and analyzing these key factors, we can better understand and address the issue of smartphone overdependence among older adults, providing a scientific basis for the formulation of relevant policies and intervention measures.

Firstly, based on the research findings, demographic factors such as age and occupation have been identified as predictors of smartphone overdependence. With the surge in smartphone ownership, the proportion of middle-aged and older adults in South Korea who are overly dependent on smart devices is also increasing year by year (Kim S. H. et al., 2024). According to NIA’s survey, in 2021, the proportion of individuals aged 50 and above at potential risk and high risk of smartphone overdependence was 14.2 and 3.5%, respectively (National Information Society Agency, 2021). While these rates are lower than those of the general population(19.7% for potential risk and 4.5% for high risk), the increase in potential risk among those in their 50s by 47.6% and by 49.6% among those aged 60 and above, compared to 2016 data (National Information Society Agency, 2016), underscores the growing concern surrounding smartphone overdependence among older adults. Additionally, occupation had been identified as a predictor of smartphone overdependence. According to the 2023 NIA survey, among individuals aged 60 and above who are employed, 11.2% are at potential risk and 3.8% at high risk, compared to 8.1 and 1.2%, respectively, among those who are unemployed (National Information Society Agency, 2023). Furthermore, studies have shown that in research examining the association between occupational status (students, employed, unemployed, or retired) and smartphone dependency, the retired group is the only group significantly associated with levels of smartphone overdependence (Linden et al., 2021). This also underscores the complex interplay of occupational factors in susceptibility to smartphone overdependence. This study, using machine learning algorithms, identified occupational factors as predictive features of smartphone overdependence among the elderly. Therefore, future research can investigate the detailed relationship between the occupational status of the elderly and smartphone overdependence.

Secondly, this study found that the primary components of smartphone usage time among older adults include communication, leisure activities, and information search and education. Several studies have shown that smartphone overdependence is associated with increased smartphone usage time (Jeong and Oh, 2020; Duke and Montag, 2017). Similar to previous studies on adolescents and university students, prolonged use of smartphones for communication is positively correlated with smartphone overdependence (Matar Boumosleh and Jaalouk, 2017; Jeong and Bae, 2022). Additionally, excessive use of smartphones for entertainment activities such as online gaming, watching videos, listening to music, and reading e-books can significantly increase overall smartphone usage time (Wang et al., 2015; Chen et al., 2017). The more time spent on social media, the stronger the inclination for immersive engagement in information search and entertainment, thereby strengthening the association between increased social media time and smartphone overdependence, which raises the risk of overdependence (Park et al., 2013; Bian and Leung, 2015). These findings indicate that, regardless of age, the duration of smartphone usage is a significant predictor of smartphone overdependence. Notably, previous evaluations of smartphone overdependence often used screen time as the primary assessment metric. However, this study adopted a more comprehensive approach by identifying the main content components of smartphone usage time through predictive analysis, rather than just screen time. This approach has significant implications for developing targeted preventive measures for smartphone overdependence.

Thirdly, self-awareness of smartphone overdependence is a predictive factor for the risk of smartphone overdependence—an aspect that has received relatively limited attention in the existing literature, thereby offering a novel contribution. According to the NIA (2023) survey, 80.9% of individuals classified as at-risk for overdependence reported that they perceived themselves as being more reliant on smartphones than others, compared to only 22.9% in the general user group—indicating a striking difference of 58.0%. Among the at-risk group, 16.4% were older adults in their 60s (National Information Society Agency, 2023), suggesting a potential misalignment between self-perception and actual usage behavior in this demographic. From the perspective of social cognitive theory, individuals’ awareness and evaluation of their own behavior plays a critical role in shaping and maintaining that behavior over time (Bandura, 1991). Self-awareness acts as a prerequisite for initiating subsequent behavioral assessment and adjustment. In the context of smartphone use, self-awareness reflects the extent to which individuals recognize the intensity and patterns of their behavior and detect early risk signals—thereby influencing their willingness and capacity to engage in corrective actions. Individuals with low levels of self-awareness may lack a clear understanding of the frequency and motivation behind their smartphone use, making it difficult to distinguish between habitual use and problematic use. This ambiguity increases their likelihood of slipping into involuntary dependency. In contrast, higher levels of self-awareness enhance individuals’ capacity to monitor their own behavior, enabling them to detect abnormal usage patterns at an earlier stage and intervene before overdependence escalates into addiction (Iskender and Akin, 2011; Park, 2019).

This mechanism is particularly salient among older adults. As social roles diminish and offline social interactions decrease with age, older individuals increasingly rely on digital media to maintain social connections. In the absence of adequate self-awareness regarding their own dependency behaviors, they may become especially vulnerable to a state of passive immersion or involuntary overuse. Thus, self-awareness in this context should not be viewed merely as a causal “foundation” for addiction prevention, but rather as a critical psychological precondition for entering a self-regulatory behavioral cycle. It is important to emphasize that the findings of this study do not suggest that self-awareness alone is sufficient to prevent or overcome smartphone overdependence. From the perspective of behavioral pathways, self-awareness should be understood as a *necessary but not sufficient* condition. Its primary role lies in facilitating the early recognition of problematic behavior and initiating self-regulatory responses. Therefore, interventions aimed at enhancing self-awareness—such as reflective feedback, guided self-monitoring, or supportive self-assessment tools—may be more effective in mitigating the risk of overdependence when integrated with broader forms of social support and digital literacy education. Such multidimensional strategies are especially critical for older populations, who face both increased exposure to digital technologies and heightened vulnerability to dependence-related risks.

Fourthly, this study identified that patterns of smartphone content usage are also predictive factors for the risk of smartphone overdependence. First, the use of smartphone communication content, including messaging, email, and social networking services (SNS), are predictors of smartphone overdependence. Research has shown that usage patterns for social purposes are closely associated with smartphone overdependence (Van Deursen et al., 2015; Jeong et al., 2016). Secondly, using smartphones to watch videos, read e-books, webtoons, and web novels, and listen to music can provide temporary enjoyment. However, as the use of smartphones for entertainment increases, psychological dependence on smartphones also intensifies. Previous studies have indicated that excessive use of smartphones for entertainment can increase smartphone addiction (Wang et al., 2015; Oh et al., 2018). These findings suggest that overuse of smartphones for entertainment not only increases dependency among adolescents and adults but also among older adults. Furthermore, the use of smartphones for information seeking, such as viewing news, searching for products/services, academic/business information, traffic and location information, and general online surfing, can increase habitual smartphone use. This increased use may heighten the risk of smartphone addiction among older adults, potentially leading to addiction (Park et al., 2013; Bian and Leung, 2015; Kim et al., 2020). Therefore, this study, through machine learning algorithms, further confirms the relationship between patterns of smartphone content usage and the risk of smartphone overdependence.

Prior research consistently demonstrates that older adults often face challenges in using digital technologies due to limited digital literacy, reduced cognitive flexibility, and a lack of confidence in operating mobile applications (Czaja et al., 2006; Choi and DiNitto, 2013). As such, targeted mobile applications—such as self-diagnostic or self-monitoring tools—may offer promising avenues for preventing smartphone overdependence among older adults. However, we also acknowledge that the effectiveness of such interventions may be constrained by practical barriers to adoption within this population. To enhance both the accessibility and efficacy of technology-based interventions, supportive strategies should be incorporated. First, simplified and age-friendly user interfaces are critical for reducing cognitive load and improving usability. Second, guidance provided by caregivers, family members, or community coordinators can assist older adults who may have limited ability to independently operate digital platforms. Third, embedding educational elements within applications or offering complementary digital literacy training can further improve user engagement and comprehension. These approaches align with broader principles of inclusive digital health design, which emphasize the importance of adapting technological interventions to the unique capabilities and real-world contexts of older users (Vaportzis et al., 2017). Therefore, future interventions aimed at reducing smartphone overdependence among older adults should not only prioritize technological innovation but also place equal emphasis on inclusivity, usability, and the provision of supportive implementation environments.

## 5 Conclusion

This research is focused on providing data-driven analysis to predict and prevent smartphone overdependence among older adults. As smartphones have become indispensable in daily life, the increasing reliance on them has exacerbated the issue of smartphone overdependence. In order to comprehensively predict the risk factors for overdependence on smartphones on older Koreans, we utilized data from the Smartphone Overdependence Survey conducted by the National Information Society Agency during 2023. Six representative machine learning algorithms were employed to identify the influencing factors of smartphone overdependence: LR, DT, NB, SVM, KNN, and XGBoost. The predictive performance was evaluated using five metrics: Accuracy, Precision, Recall, F1 Score, and AUC. Among all algorithms, the ensemble algorithm XGBoost demonstrated the highest prediction accuracy at 92.5%, while the predictive capabilities of other machine learning algorithms were lower. This confirms that ensemble learning is suitable for identifying the influencing factors of smartphone overdependence among older adults. Significant predictors identified include demographic characteristics, composition of smartphone usage time, awareness of smartphone overdependence issues, self-awareness of overdependence and coping strategies, and smartphone usage content. These findings provide valuable insights for developing targeted interventions and policies to address and mitigate the risk of smartphone overdependence in older adults.

This study demonstrated the effectiveness of using machine learning techniques to accurately assess smartphone dependence among older adults. The significance of this study lies in the ability to identify complex patterns in large datasets by applying these advanced algorithms, surpassing the limitations of traditional methods. This innovative approach not only enhances our understanding of the factors associated with problematic smartphone use behaviors but also improves data-driven decision-making strategies to address this social problem. The results of this study have important implications for practical interventions aimed at addressing excessive smartphone use among older adults. Insights derived from usage patterns can inform the optimization of targeted mobile applications, such as self-assessment or self-monitoring tools. However, it is essential to account for the real-world barriers that older adults face in adopting digital technologies—including limited digital literacy, high cognitive load, and low operational confidence.

Accordingly, future interventions should consider a hybrid model that integrates technology, education, and support. Specifically, technical tools should be complemented by educational support, assisted use through caregivers or facilitators, and age-friendly design features to enhance both accessibility and effectiveness. Furthermore, objective data generated from this study can be used to improve existing assessments and inform evidence-based guidelines to mitigate smartphone dependence more effectively. In sum, incorporating machine learning approaches into the study of smartphone overdependence offers a promising pathway toward a more comprehensive understanding of this phenomenon. It also enables the development of tailored, data-driven interventions that can better support older adults in managing their smartphone use in a healthier and more autonomous manner.

Furthermore, by utilizing the objective data obtained from this study, existing surveys could be improved and practical guidelines could be developed to effectively mitigate smartphone dependence. In conclusion, the incorporation of machine learning methods into the study of smartphone overdependence offers a promising avenue for promoting a more comprehensive understanding of this phenomenon and implementing tailored interventions to support older adults in managing their smartphone use more effectively.

This study has several limitations. First, the analysis focused exclusively on individuals in their 60s, without conducting direct comparisons across different age groups. As a result, the findings cannot be generalized to younger or middle-aged populations. Future research is encouraged to incorporate cross-generational samples to examine potential age-related differences in smartphone usage behaviors, risk perceptions, and mechanisms of overdependence. Second, individual psychological factors were not assessed as the survey relied on publicly available data. The complex relationship between smartphone overdependence and individual psychological factors deserves further exploration. Third, because this study employed a cross-sectional design, it cannot establish causality. Future research should use panel surveys to further investigate causal relationships. Finally, the dataset was drawn from a sample of older adults in South Korea. Cultural and institutional characteristics unique to the Korean context may constrain the generalizability of the findings to other national settings. For example, smartphone usage behaviors among older Koreans are shaped by distinctive societal factors, including high smartphone penetration rates, well-developed digital infrastructure, and the widespread integration of mobile services into daily life. These contextual conditions may influence not only patterns of smartphone use but also perceptions of overdependence.

Therefore, although the identified predictors offer valuable insights into understanding smartphone overdependence among older adults, caution should be exercised in extending these findings to Western or other cultural contexts, where levels of digital diffusion, social support structures, and technology norms may differ. Future cross-cultural and comparative research is needed to determine whether the predictive patterns observed in this study hold across diverse sociocultural environments and to further refine culturally sensitive intervention strategies.

## Data Availability

The datasets used and analyzed during the current study are available in the official website of NIA (https://www.nia.or.kr/site/nia_kor/main.do) and survey report (https://www.nia.or.kr/site/nia_kor/ex/bbs/List.do?cbIdx=65914). Further inquiries can be directed to the corresponding author.

## Footnotes

1
https://www.nia.or.kr/site/nia_kor/main.do
